# Publisher Correction: Detection of introduced and resident marine species using environmental DNA metabarcoding of sediment and water

**DOI:** 10.1038/s41598-020-58228-8

**Published:** 2020-02-07

**Authors:** Luke E. Holman, Mark de Bruyn, Simon Creer, Gary Carvalho, Julie Robidart, Marc Rius

**Affiliations:** 1 School of Ocean and Earth Science, National Oceanography Centre Southampton, University of Southampton, Southampton, United Kingdom; 2 Molecular Ecology and Fisheries Genetics Laboratory, School of Natural Sciences, Bangor University, Bangor, United Kingdom; 3 The University of Sydney, School of Life and Environmental Sciences, Sydney, Australia; 4 Ocean Technology and Engineering Group, National Oceanography Centre Southampton, Southampton, United Kingdom; 5 Centre for Ecological Genomics and Wildlife Conservation, University of Johannesburg, Johannesburg, South Africa

Correction to: *Scientific Reports* 10.1038/s41598-019-47899-7, published online 09 August 2019

The original version of this Article contained extensive errors in the Reference list. Reference 46 was incorrectly listed as Reference 92, and References 47-92 were incorrectly listed as References 46-91 respectively.

Additionally, there was a typographical error in the Methods.

“Records from rapid assessment surveys previously conducted for non-native invertebrates at the sample sites^47–49^ were compared with the detected species from metabarcoding data.”

should read:

“Records from rapid assessment surveys previously conducted for non-native invertebrates at the sample sites^48–50^ were compared with the detected species from metabarcoding data.”

These errors have now been corrected in the PDF and HTML versions of the Article.

